# Palliative care needs of Jordanian women’s experience of living with stroke: a descriptive phenomenological study

**DOI:** 10.1186/s12904-023-01216-2

**Published:** 2023-07-28

**Authors:** Marwa Nayef Alhalabi, Inaam Abdulla Khalaf, Ruqayya Sayed Zeilani, Hala Ahmad Bawadi, Ahmad S. Musa, Abdulqadir J. Nashwan

**Affiliations:** 1 Department of Adult Health Nursing, Faculty of Nursing, The University of Jordan, Amman, Jordan; 2 Department of Maternal and Child Health Nursing, Faculty of Nursing, The University of Jordan, Amman, Jordan; 3 Department of Adult Health Nursing, Faculty of Nursing, Al Al-Bayt University, Mafraq, Jordan; 4 Department of Nursing, Hamad Medical Corporation, P.O. Box 3050, Doha, Qatar

**Keywords:** Stroke, Women with stroke, Palliative care, Palliative care needs, Spiritual practices, Beliefs, And needs, Bothersome symptoms, Bad news

## Abstract

**Background:**

Stroke is a prevalent neurological disease that can have a profound impact on women’s physical, psychosocial, and spiritual well-being. In many cases, women living with stroke may have marginalized palliative care needs that are often not adequately addressed by healthcare providers. Unfortunately, the experience of women with stroke and their specific palliative care needs have been largely overlooked in research conducted in Jordan.

**Aim:**

The purpose of this study is to examine the specific palliative care needs of women who have experienced a stroke and are currently living in Jordan. By conducting this research, we aim to identify the various physical, emotional, social, and spiritual needs of women with stroke and gain a better understanding of how these needs can be addressed through palliative care interventions.

**Methods:**

This research utilized a phenomenological descriptive study approach to explore the experiences of twelve women recruited from the outpatient clinic of rehabilitation centers. The data was collected through semi-structured interviews. The analysis was conducted using the method of Colaizzi (1978), which involves identifying significant statements, extracting meanings, and formulating an exhaustive description of the phenomenon under study.

**Results:**

The study findings uncovered three primary themes that reflect the palliative care needs of women who are currently living with stroke in Jordan, including (1) Spiritual practices, beliefs, and needs; (2) Coping with distressing symptoms; and (3) Managing the delivery of unfavorable news.

**Discussion:**

This study provides valuable insights into the experiences of Jordanian women living with stroke, highlighting the far-reaching consequences of this condition on various aspects of their lives. The findings reveal that stroke has a significant impact on women’s physical, emotional, social, and spiritual well-being, with many facing unmet palliative care needs. By illuminating these challenges, our study underscores the importance of taking a holistic approach to stroke care that addresses the multifaceted needs of women living with stroke. Healthcare providers must consider these findings and integrate palliative care interventions into treatment plans to improve the quality of life of women living with stroke in Jordan.

**Conclusion:**

This study provides valuable insights into the palliative care needs of women who have experienced a stroke. Our findings highlight the importance of addressing women’s physical, psychosocial, and spiritual needs as part of a comprehensive approach to stroke care. We recommend integrating palliative care interventions into rehabilitation programs to improve the quality of life of women living with stroke in Jordan. By doing so, we can address the pain and complications that can arise from stroke, while also providing holistic support to address the emotional and spiritual impact of the illness. This approach has the potential to improve outcomes for women living with stroke and enhance their overall well-being.

**Supplementary Information:**

The online version contains supplementary material available at 10.1186/s12904-023-01216-2.

## Introduction

Palliative care is an approach to care that aims to enhance the quality of life for patients and their families who are facing a life-threatening illness. It involves the prevention and alleviation of suffering through early identification, comprehensive assessment, and effective management of physical, psychosocial, and spiritual needs. This includes addressing pain and other symptoms, as well as providing emotional and spiritual support to help patients and their families cope with the challenges of the illness [1]. Initially, palliative care was developed to care for cancer patients at the end of their lives, but it has developed to include care for different illnesses and can be applied early in the courses of a chronic illness [2]. Early referral of patients to palliative care facilities will help to improve the Quality of Life (QoL) for patients and their families, symptom management, decrease re-hospitalization frequencies, and reduce the costs of healthcare services [2, 3]. Many research studies investigated the palliative care needs of stroke patients as they mostly focus on the physical, psychosocial, and spiritual symptoms that develop after a stroke. Patients with stroke show debilitating physical symptoms include fatigue, constipation, dry mouth, nausea, vomiting, numbness, tingling, seizures, bladder or bowel incontinence, and seizures. In contrast, psychological and spiritual symptoms include confusion, depression, anxiety, hopelessness, and loss of meaning [4–6]. Furthermore, patients with stroke face social and cultural difficulties, due to dependence on others due to movement disorders, decreased senses, and reduced activity levels, which affect their daily lives, especially living activities such as showering, getting dressed, and going to the toilet. One of the biggest challenges for patients suffering from stroke is the feeling of pain and the inability to control and relieve it, which in turn leads to sleep disturbances and feelings of depression and anxiety [7]. Many healthcare providers are unaware of the necessity of palliative care services in caring for patients holistically from the aspect of social, emotional, and spiritual requirements, which is a significant clinical constraint [8]. The focus of clinical care has been in the traditional care of physical needs. The literature has shown a vital gap in recognizing the importance of palliative care initiated at the beginning of medical care provided to patients with stroke. It is increasingly being recognized that palliative care services should initiate at diagnosis [6].

Integrating palliative care into routine care is crucial and should not be limited to just rehabilitation services and secondary prevention programs. Palliative care should be considered throughout the entire continuum of care for patients with life-threatening illnesses, including stroke [6]. Rehabilitation programs have a crucial role in reducing physical disabilities and reducing dependence on others, while the role of palliative care arises in improving the poor and reducing the physical, psychological, and spiritual effects of stroke [9]. In addition, palliative care can provide additional resources in ensuring the management of physical symptoms, psycho-social support, and spiritual care, and helps in decision-making for patients suffering from stroke and their families [10]. Accordingly, Burton, Forster [11] highlighted the importance of conducting a thorough assessment of the palliative care needs of patients with stroke and taking appropriate steps to address those needs. By identifying the physical, emotional, and spiritual needs of patients, healthcare providers can develop a comprehensive care plan that addresses the unique challenges of each patient’s illness. Stroke survivors have been the subject of extensive research, including studies that explore their quality of life following the event [12, 13], and others exploring how stroke impacts their lives [14, 15]. A limited number of studies have specifically examined the gender differences in the effects of stroke on women’s health compared to men’s [16, 17]. Despite the extensive body of research on stroke survivors, there is still limited knowledge regarding the palliative care needs of stroke patients. This gap in understanding highlights the need for further research to explore this area, particularly among women living with stroke in Jordan. The current study aims to address this gap by examining the specific palliative care needs of women with stroke in Jordan, providing valuable insights into an understudied aspect of stroke care. By shedding light on this area, we can develop more comprehensive and effective strategies to improve the quality of life of stroke patients and enhance their overall well-being.

## Methods

### Study design

A descriptive phenomenological design utilized in this study.

### Setting

Participants were recruited from outpatient clinics and rehabilitation centers located in two major government-operated hospitals in Jordan, which are affiliated with the Ministry of Health (MOH).

### Participants

To recruit participants who were most relevant to the study’s needs and knowledgeable about the phenomenon, we used a purposive sampling approach. This method allowed us to select participants who could provide valuable insights into the palliative care needs of women living with stroke in Jordan. A total of 12 participants were included in the study, which was determined to be sufficient to achieve data saturation. Table 1 provides a summary of the demographic characteristics of the participants, which includes information on their age, marital status, educational level, and employment status pre- and post-stroke etc.

**Table 1:** Participants’ demographical characteristics

Participant no.	Age	Marital status	Number of Children	Education level	Stoke history	Employment Pre-stroke	Employment Post-stroke	Health insurance	Date of the interview
1	42	Married	7	Secondary school level	2 years	Yes	No	Public	28/6/2021
2	50	Married	7	Elementary school level	9 years	No	No	Public	8/7/2021
3	42	Married	6	Elementary school level	7 years	No	No	Public	19/7/2021
4	48	Single	----	Secondary school level	3 years	Yes	No	Public	3/8/2021
5	50	Married	4	Secondary school level	6 years	No	No	Public	21/8/2021
6	48	Widow	4	Elementary school level	3 years	Yes	No	Public	24/8/2021
7	43	Married	3	Secondary school level	7 years	No	No	Public	26/8/2021
8	29	Married	1	Baccalaureate	1 years	No	No	Public	29/8/2021
9	36	Married	2	Baccalaureate	3 years	Yes	No	Public	04/9/2021
10	26	Married	1	Baccalaureate	1 years	Yes	No	Public	09/9/2021
11	38	Married	4	Diploma	2 years	Yes	No	Public	17/9/2021
12	39	Married	5	Diploma	4 years	Yes	No	Public	20/9/2021

The selection of participants for the study followed specific inclusion and exclusion criteria. The criteria used to identify participants from the assigned rehabilitation centers were as follows: all participants had to be women who had experienced a mild to moderate stroke and were between 18 and 50 years of age, in line with the age range used in Leahy and colleagues’ study [12]; In addition to the criteria mentioned earlier, participants were included in the study if they had experienced a stroke within the last six months and were capable of comprehending and participating in a detailed interview without any major mental or linguistic disorders. These criteria ensured that the participants could provide accurate and detailed information about their experiences living with stroke and their palliative care needs. Participants with other major conditions that may overshadow the experience of strokes, such as cancer, multiple sclerosis, or renal failure, were excluded from the study.

### Data collection

The study aimed to explore the perceived palliative care needs of stroke survivors, by asking questions related to their self-perception post-stroke, ways they alleviate their suffering, physical care needs, emotional support, spiritual support, helpful care for them and their families, and future hopes. By asking these probing questions, the study aimed to gain insights into the needs and perspectives of stroke survivors regarding palliative care, to improve care services in this population.

The research team used semi-structured interviews to create a comfortable space for the participants to share their experiences and stories in a free-flowing manner. These interviews were conducted in the participants’ homes to provide a familiar and relaxed environment. The researchers collected data through 12 individual face-to-face interviews with each woman, ensuring privacy and confidentiality by not having anyone else present during the interviews. This approach enabled the participants to provide rich and detailed information about their experiences, contributing to a more comprehensive understanding of the research topic (Supplementary file 1).

### Data analysis

**Fig. 1:**
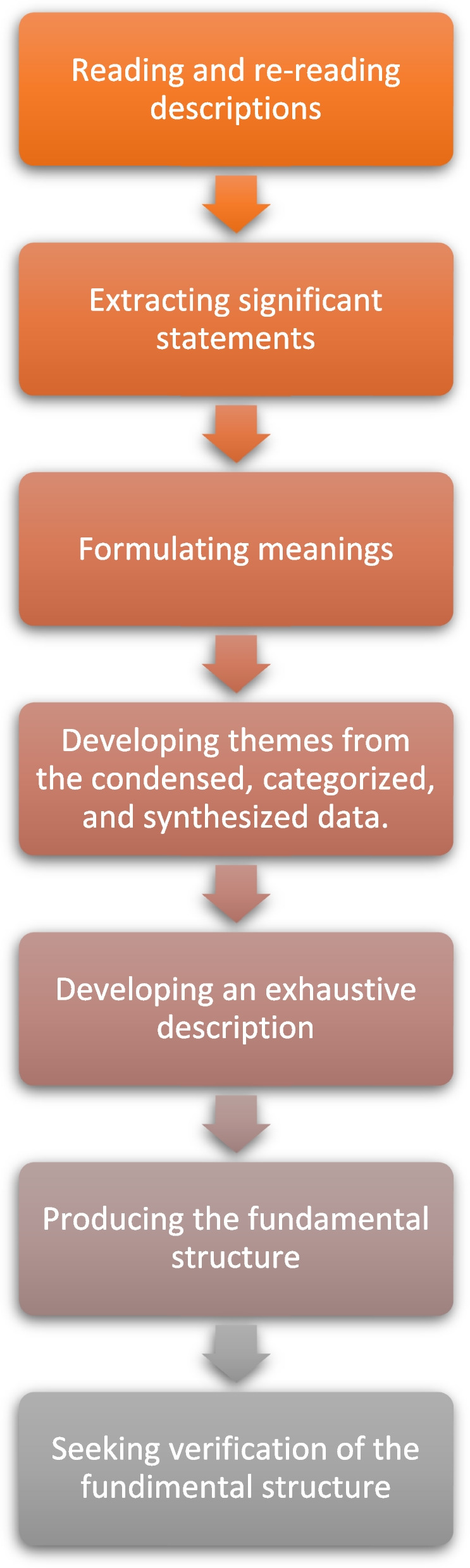
Illustrates the process of data analysis created by Colaizzi (1978)

In accordance with the Colaizzi’s method, the analysis was conducted using a seven-step process as shown in Fig. 1.

The authors used axial coding, which involves identifying relationships and connections between units of meaning formulated from each significant statement to develop broader themes. In steps four and five, the authors clustered the formulated meanings into themes that are common across all transcriptions. Then the authors wrote a full and inclusive description of the phenomenon under investigation, which is presented as a comprehensive statement as recommended by Collaizzi, (1978). In steps six and seven, the authors condensed the exhaustive description down to a short, dense statement that captures just those aspects deemed essential to the structure of the phenomenon under investigation. Finally, the authors validated the exhaustive description with each participant woman using member checking, through providing the women with the research findings via phone call, I asked them whether it captured their palliative care needs during the stroke.

The authors used the forward-translations (Arabic → English) and back-translations (Arabic → English →Arabic) methods according to WHO guidelines to ensure data integrity during the translation, interpretation, and presentation in English (WHO, 2021) [13].

### Trustworthiness

Trustworthiness is considered a gold standard for qualitative research and includes the following items, namely, credibility, dependability, conformability, and transferability; To confirm the trustworthiness, these principles were applied throughout this research study, starting by choosing the research questions and ending with the research findings. First, the researchers ensured credibility by choosing the participants from women who lived the experience and had rich data about the phenomena under study and matched eligibility criteria to capture variable perspectives regarding the specific research questions. Other strategies for improving credibility were prolonged engagement and member checking. Second, the researchers used peer debriefing and interview guidelines during each interview to ensure dependability. Third, confirmability was ensured by separating the researcher’s preconceptions, experiences, and beliefs from the descriptive raw data during all phases of the study to reduce preconceived notions and potential bias. Finally, the researchers provided a rich and thick description of the data in detail to enhance transferability.

### Ethical considerations

The ethical approval to implement the study was gained from the ethical research committee of the Ministry of Health (MOH) in Jordan, on June 20, 2021 (Ref no. 4479).

## Results

A total of 230 significant statements were identified and extracted from the data. Subsequently, the researchers organized these formulated meanings into thematic clusters. The analysis of interview transcripts revealed three key themes that captured the palliative care needs of women living with stroke: (1) Spiritual practices, beliefs, and needs; (2) Coping with distressing symptoms; and (3) Managing the delivery of unfavorable news, as presented in Table 2.

**Table 2:** Themes that emerged from the women’s perception of palliative care needs

Themes	Sub-themes
**Spiritual practices, belief, and needs.**	• Generating and sustaining hope. • Find meaning in their life and illness *“Why me?”*. • Seeking spiritual support. •
**Coping with distressing symptoms**	• Experiencing Physical impairment. • Experiencing intolerable pain. • Experiencing psychological and emotional distress.
**Managing the delivery of unfavorable news**	• The challenge when received stoke diagnosis (Shock stage). • The need for clarification and empathically response from the health care providers.

### Theme 1: Spiritual practices, beliefs, and needs

Spiritual and religious strength can play a crucial role in helping women cope after experiencing a stroke. Adequate reinforcement of this aspect can protect stroke survivors from psychological and emotional distress, providing them with the power and resilience to face life after a stroke. The findings of this study highlight that woman have spiritual needs across all dimensions, such as the need for continuous prayer and reading the Holy Qur’an, the need to maintain hope and strength, and the need to talk with religious people and those close to religion. Additionally, stroke survivors often have a need to find meaning in their illness and suffering. This theme includes three significant sub-themes: Generating and Sustaining Hope, Finding Meaning in their Illness and Suffering (“Why Me?“) and Seeking Spiritual Support. By addressing these spiritual needs and offering spiritual support, healthcare providers can help stroke survivors improve their overall well-being and quality of life.

#### Generating and sustaining hope

Almost all participants were optimistic about restoring their normal lives and striving to maintain any glimmer of hope to hold on to life. In addition, they expressed their hope that they would recover completely and regain themselves. These methods had a positive effect on their adaptation to the disease.

Participant (10) stated: “*A person with this experience can come up with a positive attitude if she continues to maintain hope and trust in God and believes in herself. I tell myself every day that I will stay well. I believe in myself that I will return to my health if I maintain a positive outlook. I will not remain using the wheelchair forever.”*

Another participant (6) indicated that the ability to maintain spiritual well-being and an optimum hope level comes from the health care provider, she said: *“Because the nurse was very supportive, I chose to commit to occupational therapy sessions rather than physical one. She would come and talk with me while I was in agony and sobbing, trying to soothe my anguish and support me. She remained with me to talk and offer me an advice, hope, and support.”*

#### Find meaning in their illness and suffering “Why me?”

The findings of this study revealed women’s perceptions and attitudes toward finding meaning in illness and suffering in their lives. Some participants refer to some positive meanings of their illness and suffering, and that their stroke is a time for reflection and enables individuals to count their blessings in life, in addition to the affliction that relieves sins and human mistakes. One of the participants (2) said in this regard: *“Illness is a test from Allah. I believe that the greater the suffering and pain, the fewer sins will inevitably be”.*

Participant (10) expressed her gratitude for being lucky enough to have a stroke to learn to be better and closer to Allah. She said: *“After surviving the stroke, I thanked Allah for giving me another chance to live. I realized that health is very important in my life. Allah gave me a chance to repent my many sins”.*

On the contrary, five participants saw that their stroke had negative meanings of their lives; they found that their illness may be Allah’s punishment for them, or that they are not righteous, and Allah is angry with them. One of the participants (6) said: *“I’m not sure why I acquired this sickness in particular; did I ever do something wrong in the past for which I’m now paying the price?”* Along the same lines, another participant (8) said: *“I am a human being, and no matter how strong my faith is, I occasionally question Allah, “Why me? Why did you put me through such a severe test? What did I do?”*

#### Seeking spiritual support

The majority of participant believed that their illness and wellness were in the hands of Allah. Hence, they accepted their illness and God’s decree. Accordingly, they maintain a close connection with God through prayers, reading the Holy Quran, and asking God (Doa’a) for recovery. Participant (2) expressed: *“In my prayers, I constantly asked God to help me regain my health. I pray to God for the patience I need to get through this hardship because I think that only God can help me solve my difficulties. Prayer has always been a source of comfort and a means for me to cope with illness.”*

According to some women, this acceptance made coping with stroke events easier for them and helped decrease suffering from unwanted consequences related to stroke. According to one of the participants (9): *The initial source of my adaptation to the new environment was my faith in God, devotion to prayers, and reading the Qur’an.”*

Many women have realized that getting closer to God and spirituality provides a sense of peace and comfort, as this has a direct impact on increasing motivation and improving their quality of life. Nine of the participants revealed a need for spiritual support, and a desire to remain connected with others who are seen as sources of spiritual support for them, whether from a religious person, family, friends, or relatives. Participant (3) stated that: *“My husband and children have always been supportive of me and have prayed passionately for my recovery. They have always been there to assist me and to help me come closer to God”.*

Despite the high degree of faith among Jordanian participants and the close connection with God, few participants thought that the cause of the stroke was the envy (Al-Hassad) or demonic acts, and for this, they resorted to religious men for healing and to help them adapt to the new situation. A participant (5) said: *“Envy is the cause of my stroke. I got it after my daughter’s marriage. Someone envied me. I think that medicine is not my treatment. For this reason, we asked my husband’s friend to treat me with non-medical methods and expel evil spirits from my body*.” Some women indicated that they resort to some traditional practices to treat the effects of stroke, such as using herbs and oils. For example, one of the participants (2) said: *“Olive oil and (Zamzam) water were the items that helped me feel better; they come from a blessed tree and have beneficial properties, and I read the Qur’an every day. I apply oil to my hands and feet and sip (Zamzam) water, which I highly suggest to everyone.”.*

### Theme 2: Coping with distressing symptoms

All participants experienced various physical, psychological, social, and spiritual challenges. This includes experiencing physical impairment, suffering from intolerable pain, Experiencing psychological and emotional distress.

#### Experiencing physical impairment

When participants were asked to describe their life experiences with the illness, all participants mentioned various types of physical impairments, such as weakness or paralysis on one side of the body, imbalance; constipation or diarrhea; difficulty chewing solid food or swallowing food; bladder or bowel incontinence. Some participants described that had very weak hands and legs to the point that they could not walk or move their bodies. In addition, to the continual discomfort and numbness. This was mentioned by participant (7), *“My leg and hand were affected, and I was nearly paralyzed. I can’t walk or move without help.”*

Fatigue was a prominent and bothersome experience. All participants mentioned that they felt fatigued most of the time. Participants described fatigue, decreased physical endurance, often leading to less energy, and feeling unable to do routine work at home. Many women had an impact on women’s everyday lives, and they had no power to serve themselves. Participant (8) said: *“Most of the time I can’t do anything; I don’t have the energy to move my body. Fatigue affects my life and always makes it depressed and boring.”*

#### Experiencing intolerable pain

Pain after stroke is a prominent shared experience among women. Most women described their experience with pain; there was some variation among the study participants regarding pain descriptions. Some participants described the pain as annoying, while others described it as terrifying. Participant (7) said: *“I’ve been in pain until now. The pain is very annoying and terrible, accompanied by a feeling of fatigue”*. While another participant said (9): *“I still feel pain after the stroke, but it is just an unpleasant feeling”.*

Most of the women reported having limitations in their lives caused by pain, as the presence of pain affected their daily activities and social interaction. This was clear in many women’s stories, and among the examples of this, the participant (3) said: *“Sometimes I have to stop doing anything because the pain increases when I try to move my hand. It hurts so badly. The pain prevents me from doing many things.”* Some of the women participants who had little independence in their daily lives said that it took longer because of the pain. Participant (4) said: *“I can do some special personal care, such as eating and brushing my teeth, but it takes longer because of my arm pain”.*

About half of the women expressed emotional reactions because of the pain. Participant (5) was pitiful about herself when pain aroused, she said: *“When my leg starts aching, I start crying without stopping”.* Some participants indicated that the pain affected their mood and socialization with others. Participant (11) said: *“I quickly became angry because of the pain, when the pain and numbness started on the left side of my body, I lost control of my mood, and I don’t want to deal with anyone.”*

Pain control is one of the most important needs expressed by women. Half of the participants suffer from intolerable pain, which has not received sufficient consideration and attention from the healthcare providers. Participant (6) said:

“The pain was not observed by anybody, and no attempt was made to alleviate it. While the physicians wished for me to improve, they did not alleviate the agony produced by exercise. I often wonder why I get hurt and my suffering worsens; I hope the staff will assess the pain because it has a significant impact on my life.”

The participants described how the pain had greatly affected their QoL, such as mood, bodily functions, sleep disorders, and physical activity. In support of that, participant added (7): *“Until today, I’ve been in excruciating discomfort. And this is what irritates me. I suffered from severe pain until now. And this is the thing that bothers me.”*

#### Experiencing psychological and emotional distress

The sudden and unexpected nature of stroke made the women either fully or partially dependent on others, without an opportunity to be prepared physically or psychologically for the onset of stroke.

Therefore, participant expressed many disturbances such as disability, embarrassment, sadness, feeling of becoming burdened, boredom, fear, and uncertainty. In this context, the participant (6) highlighted how she felt disabled and helpless after a stroke because she lost their independence and needed continuous help from others in all daily life activities, she stated: “*Because of the stroke, I am no longer able to do the parts that I enjoyed, and that makes me feel helpless. Now I am unable to engage in any of the activities that I enjoy. I am not used to relying on others. A stroke makes me feel powerless. How difficult, terrible, and unpleasant this feeling is.”*

Another participant (7) expressed her concern about what will happen in the future in the following words: *“My experience has been described as a weary, hazy trip in which we have no idea how long it will endure or what the future holds for us. Uncertainty is constant in my life.”*

Another feeling associated with the women’s post-stroke experiences, as reported by the women, is sadness. Nine women reported feeling sad because of their inability to accept physical and psychological changes caused by the stroke. They were unable to perform self-care activities independently, resulting in a sense of weakness as their role in all aspects of life had changed. One of the participants (4) said: “*I am tired, exhausted, and sad all the time. Furthermore, the effects of the stroke crushed my spirit and held me back. I am no longer optimistic, and I have stopped being content with anything.”*

### Theme 3: Managing the delivery of unfavorable news

The diagnosis of stroke is often a great shock to participant and families, and their knowledge of the disease and its consequences may be lacking. Therefore, the way this unpredicted news and details are presented, how do healthcare providers communicate with women with stroke, and how much time was devoted to speaking with them, are what was highlighted by the women participating in the study. This emergent theme consists of two subthemes: The challenge when received stroke diagnosis (Shock stage), and the need for clarification and empathically response from health care providers.

#### The challenge when receiving stroke diagnosis (Shock stage)

Through the women’s stories, inappropriate communication to deliver bad news was one of the most difficult situations the participants’ experienced. Six out of twelve women reported inappropriate communication skills from the healthcare providers in terms of how bad news was reported to them. This had a significant impact on their health and their ability to tolerate such news. Participant (3) provided an example of the sudden and bad way of conveying bad news to her that harmed her health: “*My doctor arrived on the second day of my hospitalization. When she entered the room, he inquired, “Who is (W. S)? She informed me, “You had a stroke; I am sorry for you since you are still to have a stroke.” I was shocked. “I replied: Really! What are you talking about? I had a stroke. Unfortunately, he informed me that I had a major stroke. He did not attempt to assist or alleviate my fears. On the same day, I experienced another round of sorrow. The news saddened and irritated me, and I had a second stroke later that night*.”

A group of women expressed their need to discuss with the healthcare provider their needs after receiving the diagnosis of stroke, but the amount of time devoted to this was insufficient, and they considered this as an important element in such circumstances, especially with this type of bad news. A participant (7) said: *“When I learned that I had a stroke, I wanted a lot of explanations, but nothing was clarified to me, and whenever I ask a doctor or a nurse to discuss, they don’t have time.”*

#### The need for clarification and empathically response from health care providers

All the participants had a wide range of emotional reactions when knowing about their stroke diagnosis and realizing their disabilities and complications that would accompany them for a long time. Some of them responded to the bad news with a state of silence or crying, and others with dramatic crying and entering a state of shock. The women needed to respond to these feelings through appropriate empathetic communication and find effective ways to process the emotions that are experienced by patients when a patient hears bad news.

Participant (5) said: *“The problem was that the doctor’s way of conveying the news shocked me. He gave me the news suddenly and grieved me. I did not find sympathy or support. It was very overwhelming.”*

## Discussion

This study sheds light on the experiences of Jordanian women living with stroke, providing a comprehensive view of how stroke impacts their lives. In addition, our findings highlight different mechanisms of resilience and adaptation that women use to cope with their new situation. Notably, our study revealed that spiritual and religious beliefs were among the most significant mechanisms used by women to adapt to their illnesses. This finding underscores the importance of considering the role of spirituality and religion in stroke care and highlights the potential benefits of integrating these elements into palliative care interventions.

Although Jordanian women were exposed to chronic diseases that left many disabilities and imbalances, many of the participants in this study stated that most of the disabilities they were exposed, should be accepted. and they consider the life does not have to stop there. According to a study conducted by Fearon & colleagues [14] among arab women with advanced breast cancer, they indicate that believing in Allah has been a source of comfort and reassurance because life, death, and pain are gifts from Allah, and Allah has the power to repair everything as it was, so it must respect and surrender to Allah destiny. Previous researchers have found similar findings [15, 16], where attachment to God, prayer, and faith may confer hope, optimism, energy, security, and dignity.

The current study showed that Jordanian women need hope for recovery, regardless of their medical condition. In the same context, previous studies confirmed that all patients, their situation, age, need, and hope, mainly derived from health professionals. Communication with hope helps to accept their diagnosis and improve their well-being and quality of life [16, 17].

The study findings indicate that some women who have experienced stroke struggle to accept their condition, particularly when they perceive stroke as something that only affects the elderly. As a result, they may struggle with feelings of disbelief and confusion, often asking themselves, “Why me?“ This questioning reflects their struggle to understand why they, as young women, have been impacted by this disease.

Many women reported other aspects related to spirituality such as finding meaning in disease and suffering. For Jordanian women, Islamic religious beliefs were a source of adaptation and acceptance of illness. It made them look at life in new ways. They felt that they were closer to God and acknowledged a blessing in life. Jordanian women explained that disease and fatalism “accepted God’s will” because God’s plan and illness are destiny. This is congruent with a study conducted with Jordanian women diagnosed with stage I, II, or III breast cancer [18], where the women used the spiritual sense to deal with the shock of having breast cancer and considered that If Allah loves someone, He will test their faith and patience, and true believers must accept God’s test by forgiving and giving thanks, and by enduring pain, they will receive God’s reward.

Moreover, Previous studies have found similar statements by Muslims [19–21]; these studies reported that Muslims view illness as an affliction from Allah and atone for their sins. So that created a new meaning for their purpose in life. Thus, their spirituality was strengthened through their faith and religious practices. This was not always positive, as two women failed to change due to different interpretations of fatalism. These participants explained the disease and fatalism; they believed the illness was connected with God’s punishment. Kouwenhoven, Kirkevold [22] reported that stroke is a disruptive life event that can include a person’s failure to return to normal, requiring self-redefinition and understanding of the meanings of suffering and disease.

This study provides a comprehensive view of the experiences of Jordanian women after suffering a stroke, highlighting the significant impact that stroke has on various aspects of their lives. The findings suggest that stroke’s burden has far-reaching consequences for women, impacting their physical, emotional, social, and spiritual well-being. This underscores the need for a holistic approach to stroke care that addresses the multifaceted needs of women living with stroke in Jordan. Therefore, the Jordanian women suffered from several bothersome symptoms and needs related to physical, psychosocial, and spiritual issues. Such as physical impairment, fatigue, pain, dizziness, impaired memory, imbalance, insomnia, urine incontinence, and impaired mobility, which limited the women performing basic Activities of Daily Living (ADLs), and self-care. Therefore, the women became dependent on others. These findings were consistent with the previous study’s findings [5, 23, 24].

Another prominent bothersome symptom that women reported was uncontrolled fatigue. These were significant symptoms that interfered with the QoL for Jordanian women and restricted them from performing many daily tasks. These results are consistent with previous studies [25–28]. A meta-analysis study that was conducted by Cumming, Yeo [29], revealed a significant relationship between post-stroke fatigue and gender. The fatigue reported by women was more frequent than in males; fatigue was one of the most prominent physical symptoms after stroke.

Furthermore, this study found that pain affects a woman’s QoL; women experienced anxiety, lack of sleep, fatigue, inability to move, and lack of energy because of chronic pain. Previous studies [30–32] have indicated that about a quarter of patients with stroke have long-term pain. Pain occurs soon after a stroke.

Although post-stroke pain was a common clinical problem and affected women’s QoL, it is still undiagnosed, neglected, and marginalized by health care providers as they did not receive adequate training in pain management. An integrative review conducted by Payton and Soundy [33] recommended that pain should be diagnosed and evaluated in a detailed and documented manner as soon as a patient begins to complain of pain, emphasizing the importance of effective communication between patients and caregivers to understand the patient’s experiences of pain and pain-related problems, as pain treatment is often associated with poor evaluation and understanding of pain management. While Nesbitt and Moxham [24] confirmed that there is many patients complaining of ineffective pain management, this may be related to communication disorders and cognitive problems in stroke patients.

Psychological and emotional distress is a common complication after stroke and impacts all aspects of recovery [34, 35]. This study explored many psychological symptoms experienced by Jordanian women, such as anxiety, loss of self-control, grief over life before the stroke, boredom, and social isolation. The causes of psychological distress were closely related because the participants were still at an age; they believed that they had many responsibilities and obligations that could not be accomplished with their current physical impairments. These findings were consistent with the findings of previous studies [27, 36]. Based on the shared views from all the study participants, this research identified experiences mostly focused on their emotional disturbances. Most of these feelings harmed women’s emotional and psychological health while experiencing the effects of stroke. One of the most critical issues revealed by this study is what the participants reported about their feeling of burden on their family and their sense of burden caused because of their total or partial dependence on their families to help in accomplishing daily tasks, in addition to increasing the financial burden on them. This is consistent with the results of previous studies where participants reported similar concerns [37, 38].

It is important to note that in Jordan, as in other cultures, stroke may be perceived as a significant and life-changing event, particularly for women who may be the primary caregivers in their families. Various cultural and social factors, such as religious beliefs, family values, and societal attitudes toward disabilities and health conditions, can impact the stroke experience. For example, in Jordan, family support and involvement in caregiving are highly valued, and there may be expectations for women to take on caregiving responsibilities. Additionally, attitudes towards disability may vary, and some individuals may view disability because of divine will or fate. These cultural and social factors may influence how stroke survivors and their families perceive and cope with the diagnosis and its impact on their lives. For that, the findings of the study showed that most Jordanian women faced a problem in receiving information related to their illness from healthcare providers, which was a particularly poignant topic in their life. The stroke events were shocking and unexpected, and most of the news about their condition was bad and changed their expectations and hopes for the future.

The findings are consistent with what was reported in previous studies, whereby stroke patients suffered from negative affect and emotional disturbances due to ineffective communication when bad news is communicated to them by the health care providers [39–41]. Research in the field of stroke indicates that health care providers must use effective skills in Breaking Bad News (BBN) in terms of clear style, realism, and hope, to avoid the occurrence of unwanted psychological and emotional symptoms such as shock, anger, and annoyance [42, 43]. In this study, not using a therapeutic method when telling bad news left the women in a state of confusion and shock. In the same context, a systematic review of 30 studies from eight countries indicated that to ensure emotional recovery and improve the experiences of stroke patients and their families, health staff should adopt an approach based on breaking bad news compassionately, and preparing the staff to meet the information needs of patients and their families [11].

Most Jordanian women strongly desire professional communication from healthcare providers; they describe the communication they need as caring, empathetic, compassionate, and respectful. The women’s stories about their contact with the healthcare provider reveal how words convey significant meaning, women who often look to the healthcare provider for reassurance, understanding, and support; according to Denham, Wynne [44], the professional, smooth, empathetic way in communication process consider crucial aspects in better communication between health care providers and patients living with. Previous studies found that communication between women and professionals was found to be an essential element in building a trust and confidence relationship in the care process [45–47]. Furthermore, appropriate communication has great importance in meeting the healthcare needs of women and their caregivers [48].

The women in the current study expressed the necessity of communicating bad news in an empathetic manner, in respectful, clear, and understandable language, in order not to add an additional burden to their health condition. This finding was consistent with a study conducted by Read, Heslop [49] who emphasized the importance of preventing bad behaviors during communication with stroke patients that will be reinforced their feelings of shame and unfairness.

The Jordanian women reported in this study negative behavior and attitudes about health care providers during communication, which was unsatisfactory and ineffective. The women expressed that they believe that the health care providers are the most prominent supporters and the most important pillars that help in the recovery process post-stroke. However, the expectations of most women were not fulfilled due to the way of conveying bad news to them, receiving a minimum of time and sympathy from them. The women believed that their health outcomes and mental status would have improved if the health care providers had better skills in communicating bad news and had more emotional support.

The current study findings indicated that healthcare providers need to be well experienced in proper strategies for communicating effectively with women. For example, if they were using the SPIKES protocol (SPIKES: Setting; Perception; Invitation; Knowledge; Empathy; Summarizing), which was effective in communicating bad news with different populations and different cases [50, 51]. Allowing more time for discussion with women to express feelings and answer their inquiries was recommended by Jordanian women.

### Palliative care with stroke patient

This study described the palliative care needs of patients with stroke from their perspective. The most common symptoms among participant that needed palliative care were Fatigue or lack of energy, pain, reduced physical function, dizziness, impaired memory, dependency on others, social isolation and loss of identity, depression, sexual disorders, and the need for spiritual and psychological support. In addition, a decrease in the quality of life and well-being of the woman and the caregiver was detected due to the massive change in their life roles after a stroke. The palliative care approach is closely related to the needs of women, as it is a comprehensive approach to all aspects of the human being and includes improving the quality of life for the patients and families. The World Health Organization (WHO) reported palliative care as an interdisciplinary approach to care that should be routinely integrated with disease care to improve patient and informal caregiver experience and outcomes, noting that it should be incorporated when the disease is “life-threatening” and not when the patient is also on the verge of death. It includes support to live as well as possible with poor health and bereavement care [10]. In addition, previous studies considered patients with stroke as one of the groups in need of palliative care [6, 52]. The palliative care approach to stroke patients offers a range of services to alleviate the patient’s physical and psychological suffering, improve the quality of life and the patient’s sense of living with dignity, and help the patient become equally self-reliant. In addition to providing psychological support to the patient’s family and providing more specialized services [4, 53].

Several studies have developed frameworks and models for integrating palliative care into stroke services [6, 24, 54] Steigleder and colleagues (2019) [6] identified the critical aspects of integrating palliative care into stroke care and the barriers to implementation. In addition, they provide insights into effective approaches to integrating palliative care within stroke care.

Burton and Payne (2012) [24] provide a framework for integrating palliative care and stroke care around the needs and preferences of patients and their families. The proposed framework can help clinicians and policymakers in Jordan develop a framework for integrating palliative care with stroke treatment. Accordingly, early integration of a palliative care approach besides stroke care within Jordanian health services, will prevent exacerbation of bothersome symptoms, meet psychosocial and spiritual needs, reduce hospitalization, and thereby reduced health costs burden. Moreover, palliative care will reduce suffering, increase the quality of life for patients and families, and allow patients to remain and possibly die at home under the care of their loved ones.

### Limitations

There are some limitations to be addressed in this study, the first limitation of this study is that all participants were recruited from governmental hospitals, which may limit the generalizability of the findings to stroke patients treated in other healthcare settings. Secondly, this study only focused on women’s experiences and did not assess the need for palliative care for stroke patients of both sexes at the community level. It is possible that men’s experiences and their palliative care needs may differ from those of women.

Despite these limitations, the study findings have important implications for nursing practice, education, policy, and research related to caring for women patients suffering from stroke. The study provides valuable recommendations to improve healthcare providers’ understanding of the experiences of women living with stroke and sheds light on the palliative care needs from the Jordanian women’s perspective. Our findings highlight the need for further evidence-based research to determine the palliative care needs of stroke patients and their caregivers. Additionally, future research should investigate the impact of integrating principles of rehabilitation and palliative care on a larger scale.

## Conclusions

This study described the palliative care needs of patients with stroke from their perspective. The most common symptoms among participants that needed palliative care were Fatigue or lack of energy, pain, reduced physical function, dizziness, impaired memory, depending on others, psychological disturbances, and the need for spiritual and psychological support. Moreover, stroke survivors may experience social isolation and discrimination because of cultural and social attitudes towards disabilities and health conditions in Jordan, additionally, patients with stroke may face challenges accessing healthcare and rehabilitation services, due to societal and structural barriers. These issues can contribute to stigma and a negative perception of stroke in Jordan. However, it is important to note that stroke can also be viewed positively as an opportunity for personal growth and resilience. Awareness-raising campaigns and support groups can help reduce stigma and improve outcomes for stroke survivors. The use of appropriate techniques to deliver bad news health care providers is crucial for participant. Accordingly, early integration of the palliative care approach besides stroke care within Jordanian health services, will prevent exacerbation of bothersome symptoms, meet psychosocial and spiritual needs, reduce hospitalization, and thereby reduced the health cost burden. Moreover, palliative care will reduce suffering, increase the quality of life for patients and families, and allow patients to remain and possibly die at home under the care of their loved ones.

## Supplementary Information

**Additional file 1. **Interview guide.

## Data Availability

All data generated or analyzed during this study are included in this published article.
