# Supplementary material for: Palliative care needs of Jordanian women’s experience of living with stroke: a descriptive phenomenological study
Source: BMC Palliat Care. 2023 Jul 28;22:106. doi: 10.1186/s12904-023-01216-2 (PMC10375733; doi:10.1186/s12904-023-01216-2)
Supplement: Supplementary file 1 — Additional file 1. Interview guide. [file 12904_2023_1216_MOESM1_ESM.docx]

**Interview guide**

Study main question:

- What are your perceived palliative care needs during suffering from a stroke?

Then followed by the following sub-questions?

- 1. Has stroke affected the way you see yourself?
  2. How do you relieve your suffering?
  3. What special care do you need for physical care?
  4. What special care do you need for emotional support?
  5. What special care do you need for spiritual support?
  6. What would other care be helpful to you and your family?
  7. What are your hopes for the future?
